# Research on a driving risk model for urban tunnel

**DOI:** 10.1371/journal.pone.0345766

**Published:** 2026-10-09

**Authors:** Song Fang, Wenting Lu, Baoliang Wang, Ting Yang

**Affiliations:** 1 Jiangsu Vocational College of Electronics and Information, Huaian, China; 2 Jiangsu Provincial Department of Industry and Information Technology, Nanjing, China; Henan Polytechnic University, CHINA

## Abstract

This research introduces a novel driving risk assessment framework for urban tunnel environments, founded upon the physical principles of field theory. The framework conceptualizes three distinct but interacting fields: the kinetic energy field, behavioral field, and potential energy field. The study subsequently elucidates the evolutionary dynamics of the driving risk field, which is precipitated by the presence of neighboring vehicles, individual driving behavior patterns, and alterations in the roadway environment. A comprehensive driving risk field model for urban tunnels was constructed, and the variation patterns of the driving risk field forces acting on vehicles were examined. The results indicate the magnitude of the driving risk field force is inversely proportional to inter-vehicle spacing and the distance to the tunnel sidewall, while directly proportional to the relative speed between vehicles and the longitudinal slope gradient. Under identical conditions, the driving risk field force acting on vehicles at tunnel entrances and exits is greater than inside the tunnel, and the influence of speed on the risk field force exceeds that of distance. The proposed model effectively describes the variation trends of driving risks during vehicle travel in urban tunnels, and the outcomes of this research contribute to laying a theoretical groundwork for optimizing the planning, design, and management protocols of urban tunnels.

## 1. Introduction

The roadway traffic system is best characterized as a complex dynamical system, an emergent phenomenon arising from the tight coupling and continuous interactions among four core components: the driver, the vehicle, the roadway infrastructure, and the ambient environment. The reliable and safe operation of this system is fundamentally contingent upon the reliability of each constituent and the efficacy of their synergistic integration. Among the four fundamental elements, the driver plays a dominant role by receiving state information from other elements through sensory organs, processing it through judgment, and making decisions to control the vehicle’s movement. The significant variations in urban tunnel environments may cause drivers’ physiological and psychological indicators to exceed critical thresholds, leading to abrupt changes in driving behavior and consequently generating high-risk zones. Vehicles operating in tunnel sections typically exhibit noticeable processes of speed reduction for adaptation, stable operation, and acceleration to exit. Excessive speed differences between vehicles may create possibilities for loss of control or rear-end collisions.

An analysis of tunnel traffic accident data consistently identifies rear-end collisions as the most prevalent incident type, wherein speeding constitutes the root cause as the predominant contributory factor. Speeding behavior in tunnels is primarily caused by drivers’ visual illusions. Visual illusion refers to situations where observers receive insufficient or delayed visual information when observing objects, leading the brain to construct and fill gaps based on life experiences or inappropriate visual references, resulting in visual images that deviate from objective reality. Influenced by objective conditions and personal psychological factors, observers develop erroneous perceptions and judgments of observed objects. Due to the monotonous environment, confined space, and low illumination in tunnels, drivers are prone to visual illusions and fatigue in such driving conditions, resulting in delayed responses to road information and unconscious speeding behavior, creating potential traffic hazards.

Driving risk refers to the uncertainty of losses during vehicle operation on roads, encompassing both the probability and severity of traffic accidents. The proposed driving risk field serves as a metric function formulated to quantify the aggregate impact of driver, vehicle, and roadway factors on the level of risk encountered during travel. This conceptualization is grounded in the principles of physical field theory, wherein a field constitutes a fundamental modality of material presence. In physics, a field originates from a source and can be mathematically represented by spatiotemporal functions, with its intensity exhibiting an inverse relationship with the distance from that source. By analogy, the constituent elements of a traffic system can be construed as analogous risk sources. Consequently, the magnitude of driving risk emanating from these sources also demonstrates a decaying relationship with increasing distance. An additional parallel lies in their directional nature; akin to physical fields, driving risks possess vector properties, meaning both magnitude and direction are integral to their characterization. It is by virtue of these shared attributes that the application of field theory to the domain of driving risk analysis is both justified and theoretically sound.

The structure of this paper is as follows. Following this introduction, Section 2 commences with a review of pertinent literature. Subsequently, Section 3 delineates the framework for model development. The final section synthesizes the discussion and presents the concluding remarks.

## 2. Literature review

Tunnels, as critical components of transportation infrastructure, pose distinctive challenges to driving safety due to their enclosed structure, dynamic lighting conditions, and unique geometric design. With the continuous expansion of tunnel networks and the frequent occurrence of accidents in such environments, developing a scientific and effective risk assessment model for tunnel driving has become a key research focus in traffic safety [1–3].

(1) Prevailing Frameworks for Risk Modeling

Existing research can be categorized into several theoretical frameworks. First, studies based on traditional traffic flow theory include the work of Ying Yan et al. (2019) [4], who applied the AASHTO braking model and convex hull algorithm to predict critical safe speeds. Second, potential field theory has been adopted in several studies, such as that of Haitian Tan et al. (2022) [5], which introduced a driving risk model grounded in risk field theory. Third, behavioral science approaches have been utilized; for example, Li Wan et al. (2023) [6] constructed a driving behavior profile by integrating multidimensional physiological indicators, including electroencephalography (EEG), heart rate, and eye movement data.

(2) Multi-dimensional Risk Quantification and Assessment Techniques

Researchers have established multidimensional risk indicator systems from diverse perspectives. Shuyi Chen et al. (2023) [7] defined four evaluation dimensions—lateral stability, longitudinal stability, car-following risk, and lane-changing risk—and incorporated psychophysiological states to quantify traffic safety risks. Methodological approaches are varied: Weiwei Qi et al. (2020) [8] developed a fusion model using mean pupil diameter and heart rate growth rate; Jianqiang Wang et al. (2020) [9] combined Naturalistic Driving Studies (NDS) with Driver Attitude Questionnaires (DAQ); and Yingying Xing et al. (2013) [10] built a tunnel simulation model using VISSIM software. Furthermore, Yi-Yi Ma et al. (2017) [11] employed driving simulators to assess safety in urban river-crossing tunnels. In terms of analytical techniques, Xiaoyu Cai et al. (2025) [12] applied the K-means clustering algorithm, achieving a classification accuracy of 92%; Yanli Ma et al. (2019) [13] utilized dynamic Bayesian networks; Xiao-xia Xiong et al. (2018) [14] adopted a Markov chain model for risk state prediction; and Shuyi Chen et al. (2023) [7] determined indicator weights using the CRITIC method.

(3) Model Performance and Methodological Advances in Related Fields

Recent studies indicate that modern risk assessment models, particularly those leveraging machine learning, achieve high recognition accuracy [15–17]. For instance, Jieyu Zhu et al. (2021) [18] proposed a multi-vehicle interaction risk assessment method, reporting recognition accuracies of 94.24%, 85.82%, 84.16%, and 79.69% for slight, low, medium, and high risk levels, respectively. Through structural equation modeling, Naikan Ding et al. (2019) [19] found that speed and distance risk perception accounted for 27% of the variance in collision risk. Furthermore, Renfei Wu et al. (2024) [3] introduced a dynamic risk scoring (DRS) model capable of real-time risk level assessment, while Lin-heng Li et al. (2022) [20] advanced the theoretical framework by developing a dynamic driving risk potential field model that incorporates the effects of acceleration and steering angle.

Concurrently, methodological progress in related transportation research domains offers broader perspectives and tools for tunnel risk assessment. For example, Yang et al. (2025) [21] investigated the resilience of urban integrated transportation networks, systematically clarifying its concept, evaluation metrics, and enhancement measures—a framework applicable to tunnel network safety management. Yan et al. (2025) [22] analyzed drivers’ mandatory lane-merging decision-making behavior by constructing spatiotemporal risk indicators, providing a methodological reference for examining driver behavior in tunnel bottleneck zones. In analytical techniques, studies such as Xie et al. (2024) [23], who applied Classification and Regression Trees to predict incident duration, and Hao et al. (2025) [24], who developed a knowledge graph-based framework to quantify the impact of hazardous driving behaviors, demonstrate the deepening application of data-driven methods in traffic safety. Furthermore, Pan et al. (2025) [25] utilized a multi-source accident dataset from vehicles equipped with autonomous driving systems to explore the relationship between manual takeover and crash severity, highlighting the importance of high-resolution behavioral and contextual data. Methodologically, Li et al. (2026) [26] proposed the correlated random parameters accelerated failure time model with heterogeneity in means and variances, designed to uncover correlations and heterogeneity among influencing factors, which could provide a more nuanced analytical tool for tunnel risk modeling.

In summary, research on tunnel driving risk assessment has established a relatively comprehensive theoretical and methodological foundation, with notable progress in risk identification accuracy and real-time evaluation. With the continued advancement of artificial intelligence and data science technologies, future tunnel risk assessment models are expected to become more intelligent, adaptive, and capable of integrating insights from diverse methodological approaches. The growing trend toward fusing multimodal data—such as visual, physiological, and behavioral information—will facilitate real-time risk warning and proactive safety interventions, thereby providing more robust scientific support for tunnel traffic safety management [27, 28].

## 3. Model development

Traffic risk is defined as the uncertainty of losses incurred by vehicles during their operation on the road, encompassing the probability of road accidents and the severity thereof. The characteristics of driving risks bear a remarkable resemblance to those of fields. Within the transportation system, the four elements of human, vehicle, road, and environment all have the potential to give rise to driving risks. These risks not only exist objectively but also exhibit temporal and spatial characteristics akin to fields. A field is generated by a field source and can be represented by a spatial function. The value of the field is inversely proportional to the distance from the field source. Likewise, driving risks are generated by the various components of the transportation system. When each component of the transportation system is regarded as a risk source, the risk is inversely proportional to the distance from the risk source. Furthermore, both fields and driving risks are vector quantities when direction is taken into account. Hence, the field theory can be considered for application in the research of driving risks.

The architecture of the urban expressway tunnel driving risk field model is articulated through three constituent fields: a) the Behavioral Field, governed by driver-related factors; b) the Kinetic Energy Field, governed by the dynamics of moving objects; and c) the Potential Energy Field, governed by fixed obstacles in the road environment. The combined effect of these field energies constitutes the urban tunnel driving safety risk field intensity.

### 3.1. Kinetic energy field modeling

(1) Kinetic Energy Field Model Composition

Conceptualized as the collision risk arising from interactions with moving objects, the kinetic energy field’s intensity is characterized by an exponential function. This vector’s attributes are dictated by the states of moving objects and prevailing road conditions. The severity of the collision is positively correlated with the equivalent mass size. That is, the greater the equivalent mass of the moving object, the higher the severity of the vehicle collision. The possibility of a collision is related to the distance between the moving object and the vehicle. This article assumes that the possibility of a collision is related to the distance between the two objects as an exponential function. The degree of danger of a collision between a moving object and a vehicle is related to the direction and speed at which both are traveling. Analogous to formulae in physics, the field intensity at a point $\left(x_{b},y_{b}\right)$, generated by a source object a at point $\left(x_{a},y_{a}\right)$, is expressed as:


$$\begin{array}{c} \vec{E_{D_{ab}}}=\frac{k_{\text{1}}R_{a}M_{a}}{{\left|x_{b}-x_{a},y_{b}-y_{a}\right|}^{k_{\text{2}}}}\cdot \frac{\left(x_{b}-x_{a},y_{b}-y_{a}\right)}{\left|x_{b}-x_{a},y_{b}-y_{a}\right|}exp\left[k_{\text{3}}v_{a}cos\left(\theta_{ab}\right)\right] \end{array}$$ (1)

Where, $R_{a}$ – environmental factor at point $\left(x_{a},y_{a}\right)$;

$M_{a}$ – effective mass of object a;

$\left(x_{b}-x_{a},y_{b}-y_{a}\right)$ – distance vector between moving object and surrounding point;

$v_{a}$ – velocity of moving object a;

$\theta_{ab}$ – angle between moving object a and the distance vector;

$k_{i}$ – undetermined coefficients, all greater than 0, i=1,2,3.

In Formula (1), ${\left|x_{b}-x_{a},y_{b}-y_{a}\right|}^{-k_{\text{2}}}$ indicates that the driving risk generated by the moving object on the vehicle has a power function relationship with the distance between them. $exp\left[k_{\text{3}}v_{a}cos\left(\theta_{ab}\right)\right]$ indicates that the driving risk generated by the moving object on the vehicle has an exponential relationship with the vehicle speed and their movement directions. When two vehicles are moving toward each other head-on, i.e., $\theta_{ab}={\text{0}}^{\circ },cos\left(\theta_{ab}\right)=1$, the driving risk is maximum. When moving in the same direction at the same horizontal position, i.e., $\theta_{ab}=180^{\circ },cos\left(\theta_{ab}\right)=-1$ the driving risk is minimum.

(2) Equivalent Mass

The magnitude of risk posed by a moving object to a vehicle on the road is related to the moving object’s own mass, attributes, movement speed, etc. After comprehensively considering these factors, the equivalent mass is uniformly used to describe the effective mass. The equivalent mass of moving object a is defined as follows:


$$\begin{array}{c} M_{a}=M_{a}\left(m_{a},t_{a},v_{a}\right)=m_{a}t_{a}\left(1+\sum_{i}\alpha_{i}v_{a}^{\beta_{i}}\right) \end{array}$$ (2)

Where, $m_{a}$ – mass of moving object a;

$t_{a}$ – type of moving object a;

$v_{a}$ – velocity of moving object a;

$\left(1+\sum_{i}\alpha_{i}v_{a}^{\beta_{i}}\right)$ – velocity polynomial.

(3) Road Influence Factor

The magnitude of driving risk directly covaries with roadway conditions; specifically, the risk coefficient escalates as critical parameters—including adhesion coefficient, gradient, curvature, and visibility—become compromised. These influencing factors are uniformly described using the road condition influence factor $R_{a}$, defined as:


$$\begin{array}{c} R_{a}=R_{a}\left(n_{a},f_{a},q_{a},p_{a}\right)={\left(\frac{n_{a}}{n^{*}}\right)}^{k_{\text{1}}}{\left(\frac{f_{a}}{f^{*}}\right)}^{k_{\text{2}}}exp\left[{\left(q_{a}-q^{*}\right)}^{k_{\text{3}}}+{\left(p_{a}-p^{*}\right)}^{k_{\text{4}}}\right] \end{array}$$ (3)

Where, $n_{a}$ – visibility of the road where object a is located;

$n^{*}$ – standard visibility;

$f_{a}$ – road adhesion coefficient of the road where object a is located;

$f^{*}$ – standard road adhesion coefficient;

$q_{a}$ – road curvature of the road where object a is located;

$q^{*}$ – standard road curvature;

$p_{a}$ – longitudinal slope gradient of the road where object a is located;

$p^{*}$ – standard longitudinal slope gradient;

$k_{i}$ – undetermined coefficients, $k_{\text{1}},k_{\text{2}}<0$; $k_{\text{3}},k_{\text{4}}>0$.

Under the assumption of constant-speed tunnel traversal, the kinetic energy field varies spatially due to differing road conditions. Fig 1 illustrates the computed field intensity distribution for a vehicle within the main tunnel section.

**Fig 1:**
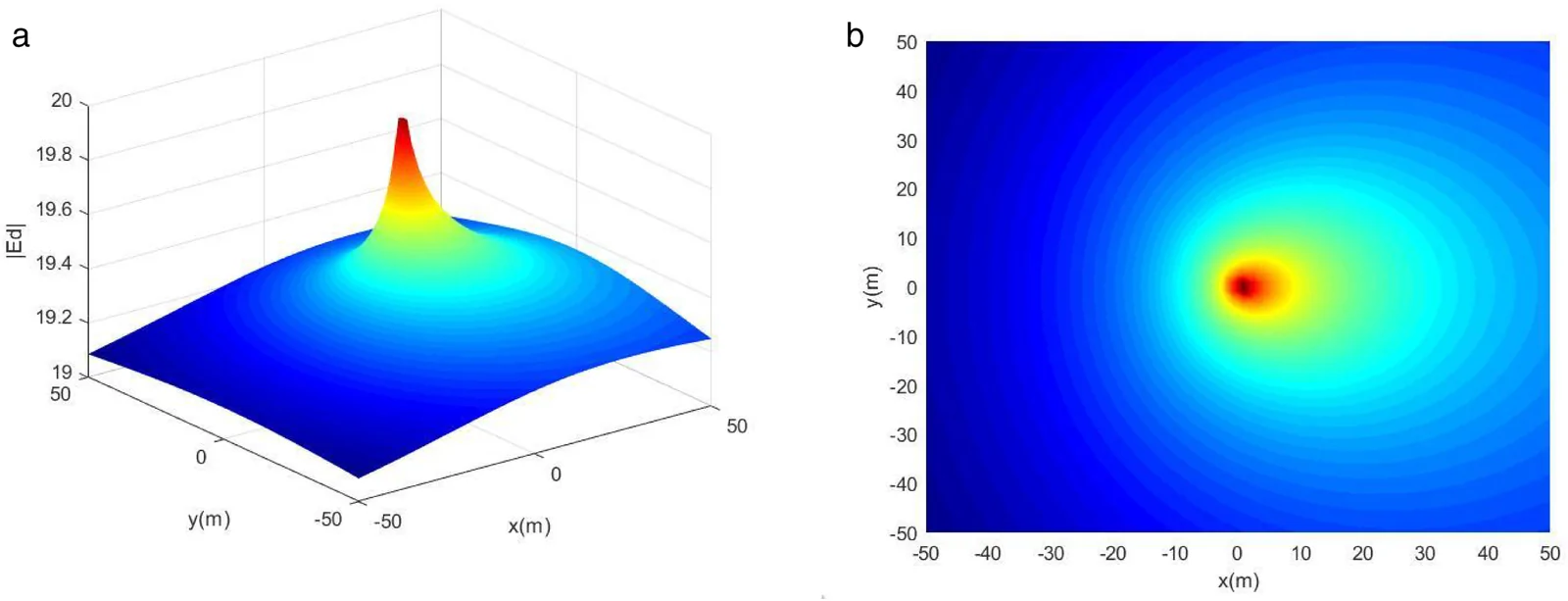
Kinetic energy field diagram in tunnel. (a) Space diagram. (b) Planform.

Fig 1 plots the kinetic energy field intensity (z-axis) against longitudinal displacement (x-axis) in the direction of travel and lateral displacement (y-axis) perpendicular to it. It can be observed from the figure that the field intensity at the kinetic energy field center (0,0) is infinite, indicating that a traffic accident occurs when another object coincides with this point. The top view in Fig 1(b) reveals that at the same distance, the field intensity value corresponding to points ahead in the vehicle’s traveling direction is relatively larger, indicating that the traveling vehicle poses a greater driving risk to vehicles ahead. The field distribution in the entrance transition section is qualitatively analogous to that inside the tunnel (Fig 2), sharing the same fundamental spatial trend. However, due to considerations of factors such as the longitudinal slope at the tunnel entrance and visibility changes caused by illumination, the kinetic energy field intensity value in the tunnel entrance transition section under the same conditions is greater than that inside the tunnel.

**Fig 2:**
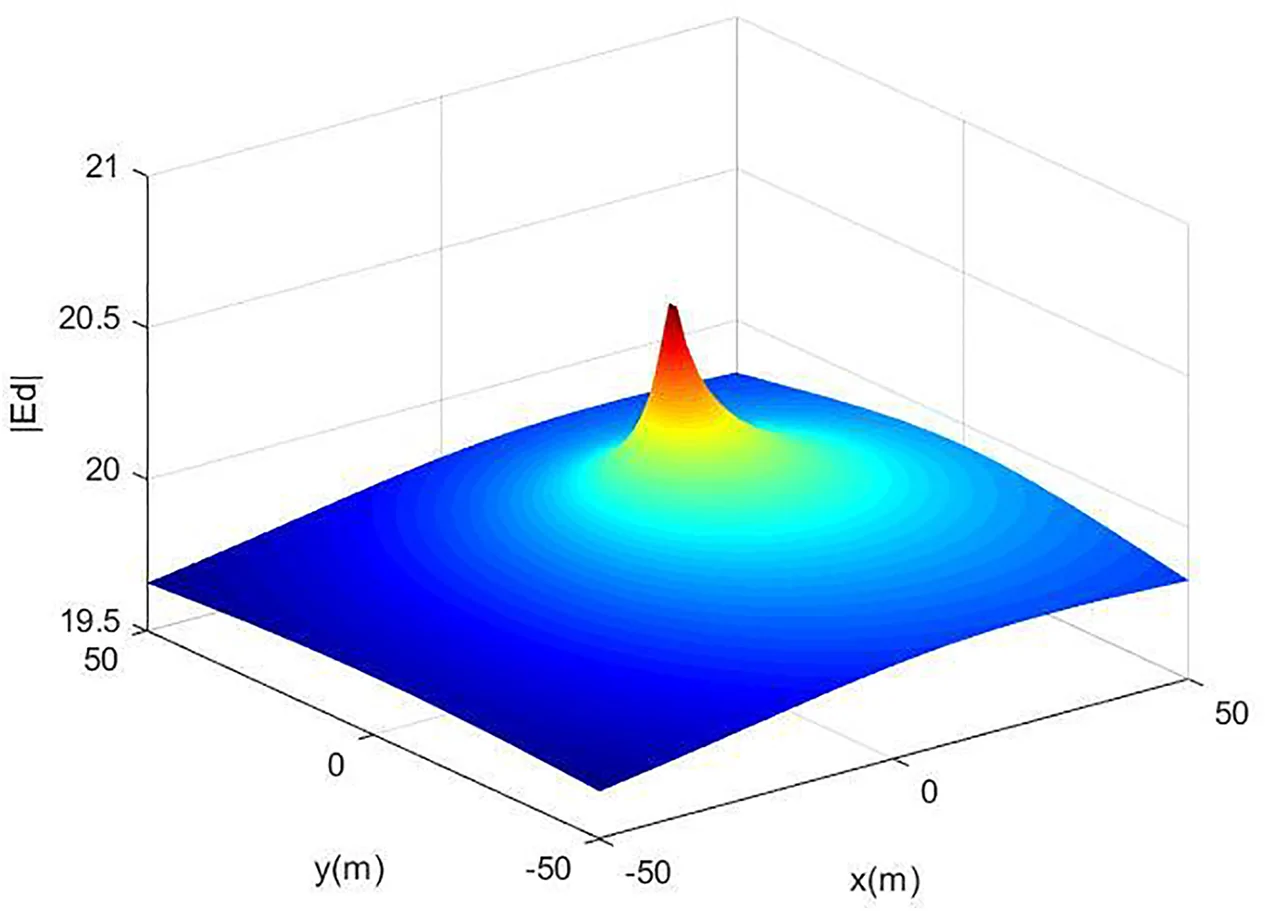
Kinetic energy field diagram at tunnel entrance transition section.

### 3.2. Behavioral field modeling

(1) Behavioral Field Model Composition

The behavioral field describes the driving behavior risk generated by driver factors during travel and the potential danger degree posed by the driven vehicle to the surrounding environment. Mathematically, the behavioral field generated by a driver is represented by the product of their risk factor and the kinetic energy field of their vehicle. The functional representation of the behavioral field intensity generated by the driver of vehicle a at point $\left(x_{a},y_{a}\right)$ at point $\left(x_{b},y_{b}\right)$ is as follows:


$$\begin{array}{c} \vec{E_{X_{ab}}}=\vec{E_{D_{ab}}}\cdot D_{ra} \end{array}$$ (4)

Where, $E_{X_{ab}}$ – field intensity of the behavioral field formed by the driver of vehicle a at point $\left(x_{b},y_{b}\right)$;

$E_{D_{ab}}$ – field intensity of the kinetic energy field formed by vehicle a at point $\left(x_{b},y_{b}\right)$;

$D_{ra}$ – risk factor of the driver of vehicle a.

The magnitude of vector $E_{X_{ab}}$ represents the magnitude of the behavioral field formed by vehicle a at point $\left(x_{b},y_{b}\right)$. A larger value indicates a higher potential danger degree, and the field intensity direction is the same as that of $E_{D_{ab}}$.

(2) Behavioral Field Driver Risk Factor

The causes of traffic accidents attributable to drivers can be classified into the following four categories: 1) physiological and psychological factors; 2) cognitive factors; 3) driving skill factors; 4) traffic violation factors.

Under constant-speed tunnel traversal, the behavioral field intensity exhibits spatial heterogeneity due to variations in driver risk factors—attributable to differing reaction times across the entrance, interior, and exit zones—and positional road influence factors. Fig 3 illustrates the resultant behavioral field distribution, which peaks at the driver’s location (0,0). A consistent trend is observed across all sections, with intensity diminishing sequentially from the entrance transition zone to the exit transition zone and finally to the tunnel interior. This intensity gradient aligns with empirical accident statistics, wherein incident frequency is highest at the entrance, lower at the exit, and lowest within the tunnel interior.

**Fig 3:**
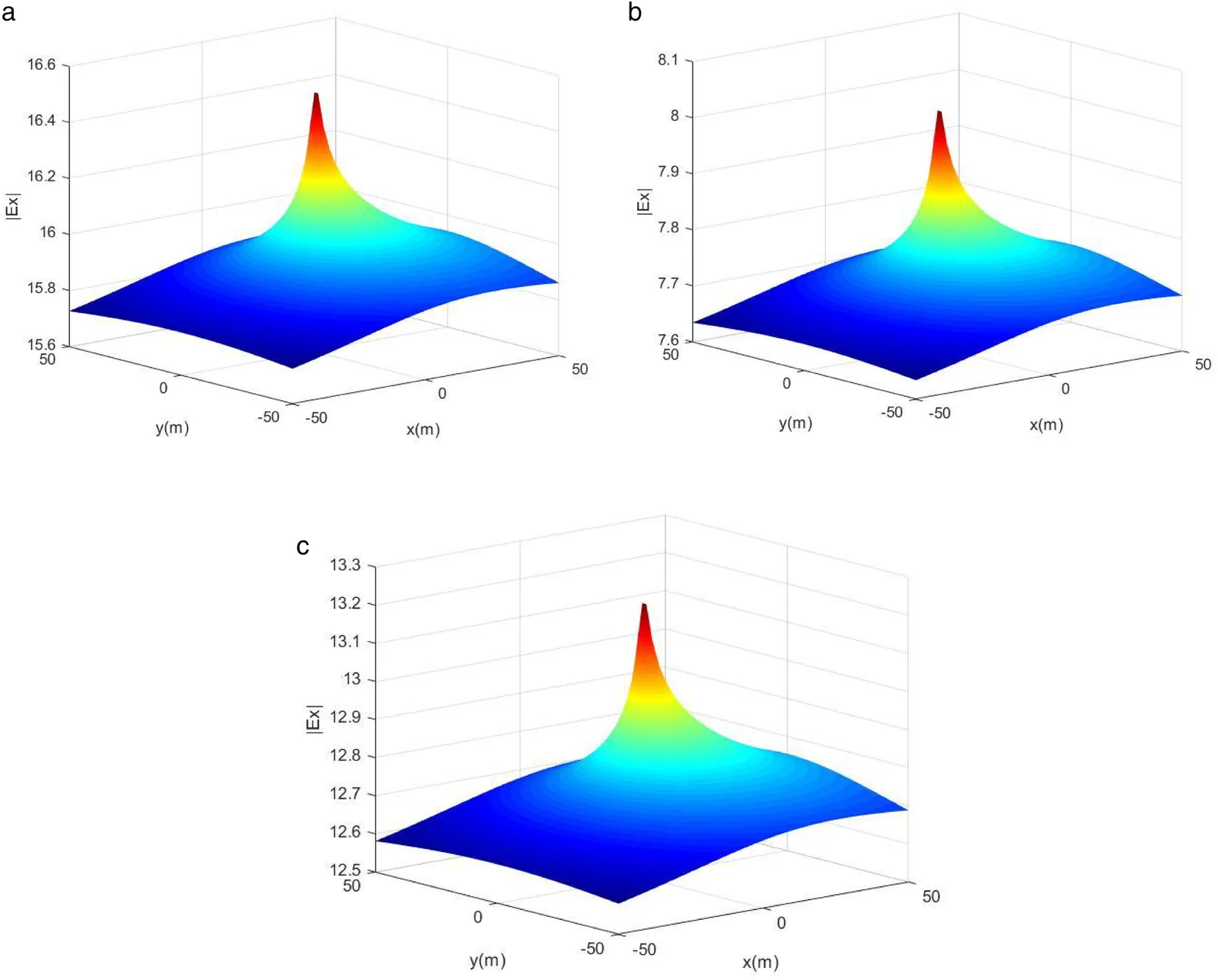
Behavior field diagram. (a) Tunnel entrance transition section. (b) Inside the tunnel. (c) Tunnel exit transition section.

### 3.3. Potential energy field modeling

The potential energy field describes the potential risk impact of stationary objects on vehicle operation, such as the gradually rising sidewalls at urban tunnel entrance and exit transition sections, central barriers, and tunnel sidewalls inside the tunnel. The extent of damage caused by a vehicle collision is directly proportional to the equivalent mass of a stationary object. The possibility of a vehicle colliding with a stationary object is related to the distance between them. The risk of traffic accidents caused by vehicles approaching a stationary object from any direction is the same. The field intensity characteristics generated by the potential energy field are similar to those of the kinetic energy field, both related to the distance between the two objects and the equivalent mass. The calculation formula for the field intensity of the potential energy field formed by a stationary object a at $\left(x_{a},y_{a}\right)$ at its surrounding point $\left(x_{b},y_{b}\right)$ is as follows:


$$\begin{array}{c} \vec{E_{S_{ab}}}=\frac{k_{i}R_{a}M_{a}}{{\left|x_{b}-x_{a},y_{b}-y_{a}\right|}^{k_{\text{2}}}}\cdot \frac{\left(x_{b}-x_{a},y_{b}-y_{a}\right)}{\left|x_{b}-x_{a},y_{b}-y_{a}\right|} \end{array}$$ (5)

The meanings of the parameters are the same as described above.

A representative urban tunnel section is modeled with the following geometry: a 600 m interior, flanked by 100 m entrance and exit transition sections each with a 3% longitudinal grade. The approach and departure road segments are each 100 m and level. Figs 4–6 depict the potential energy field intensity distributions generated by the longitudinal slopes and sidewalls within these transition zones, assuming an otherwise unobstructed tunnel.

**Fig 4:**
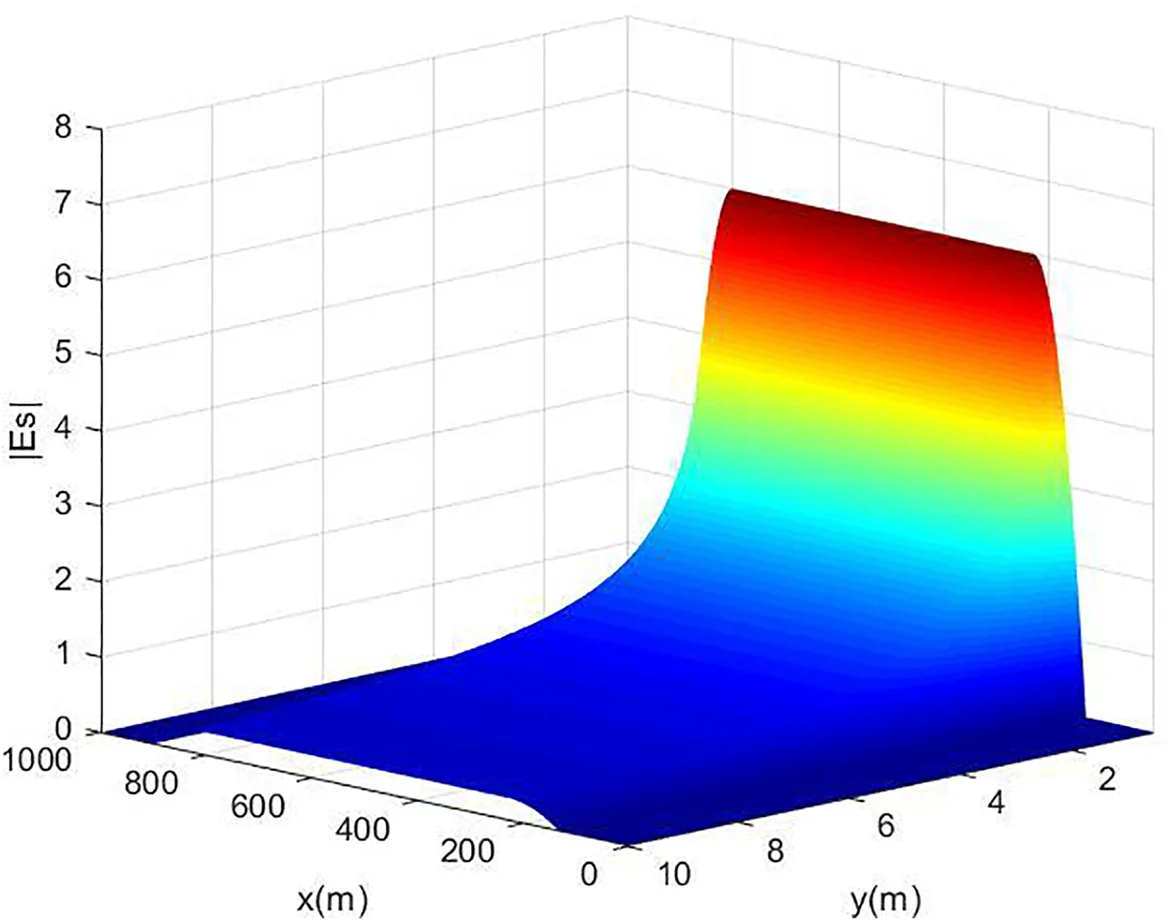
Diagram of potential energy field of tunnel sidewall.

**Fig 5:**
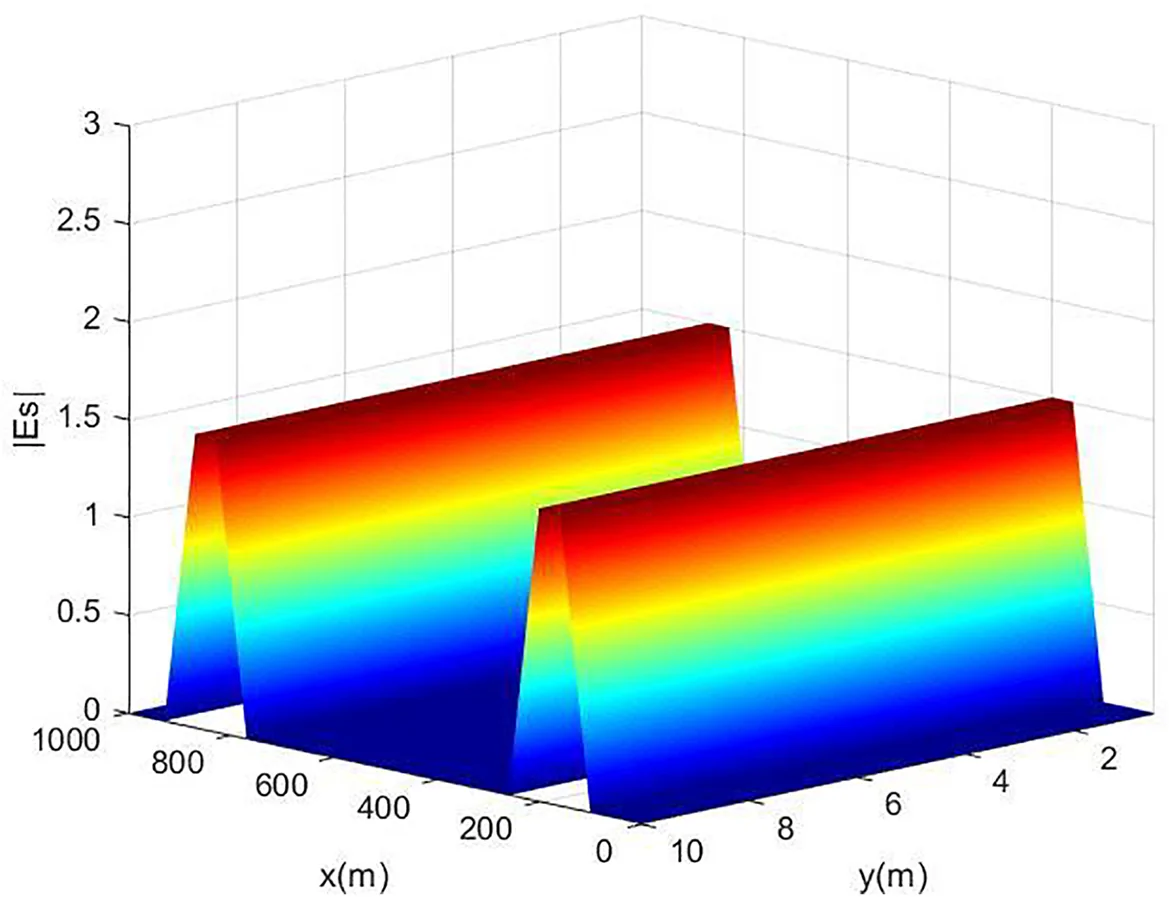
Diagram of slope potential energy field at tunnel entrance and exit.

**Fig 6:**
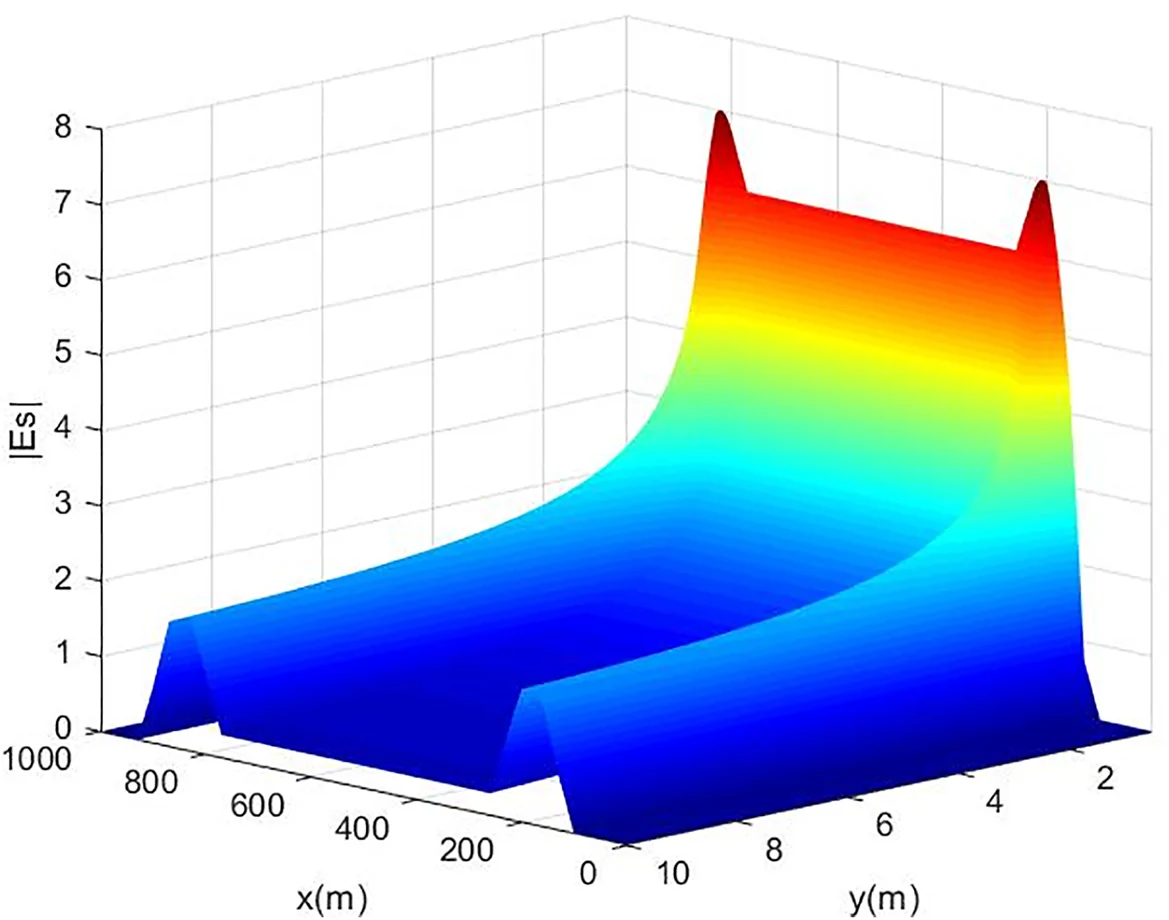
Diagram of comprehensive potential energy field of tunnel slope and sidewall.

The potential energy field exhibits spatial variation due to the non-uniform geometry of the tunnel portal regions, where sidewall height increases along the entrance transition and decreases along the exit. Fig 4 isolates the field from the sidewall alone, Fig 5 from the longitudinal slope alone, and Fig 6 synthesizes both. The x-axis denotes longitudinal position (0-100m: approach; 100-200m: entrance; 200-800m: interior; 800-900m: exit; 900-1000m: departure). The y-axis indicates lateral distance from the sidewall, and the z-axis represents field intensity. Fig 6 confirms that the maximum field intensity occurs at the tunnel portals, resulting from the synergistic effect of the slope and sidewall. This superposition induces the highest driving risk in these transition sections. The field symmetry between entrance and exit arises from the exclusion of behavioral factors. Notably, field intensity escalates nonlinearly as the y-value decreases (proximity to the sidewall increases), accurately capturing the critical collision risk, which aligns with real-world safety dynamics.

### 3.4. Driving risk field model

Within the composite driving risk field—a synthesis of the behavioral, kinetic energy, and potential energy fields—a vehicle experiences a resultant force vector. The magnitude of this force quantifies the immediate danger level, serving as a metric for both accident probability and potential severity. This vector is principally governed by the local risk field intensity, roadway conditions, vehicle properties and dynamics, and driver behavior. The greater the field strength of the driving risk area is, the greater the force acting on the vehicle will be, and the higher the driving risk will be. The more adverse the road environment conditions are, the greater the possibility of a vehicle collision will be, and thus the greater the force acting on the vehicle will be. The greater the equivalent mass, the greater the degree of vehicle collision and the damage after the collision, and the greater the force generated. When the driving speed is opposite to the direction of the driving risk field, as the driving speed increases, the possibility of a collision increases, resulting in an increase in the force acting on the vehicle; conversely, if the driving speed decreases, the possibility of a collision decreases, and the force of the driving risk field acting on the vehicle becomes smaller.

The force $F_{a}$ acting on vehicle a located at point $\left(x_{a},y_{a}\right)$ in the driving risk field can be expressed as follows:


$$\begin{array}{c} \vec{F_{a}}=\vec{E_{a}}R_{a}M_{a}exp\left[-k_{\text{3}}v_{a}cos\left(\theta_{ab}\right)\right]\left(1+D_{ra}\right) \end{array}$$ (6)

Where, $\vec{E_{a}}$ – driving risk field intensity of vehicle a at point $\left(x_{a},y_{a}\right)$;

$R_{a}$ – tunnel environmental condition at point $\left(x_{a},y_{a}\right)$ for vehicle a;

$M_{a}$ – equivalent mass of vehicle a;

$v_{a}$ – velocity of vehicle a;

$\theta_{ab}$ – angle between the velocity direction of vehicle a and the field intensity $E_{b}$ direction;

$D_{ra}$ – driver risk factor of vehicle a.

Vector $\vec{F_{a}}$ represents the driving risk field force acting on vehicle a at location $\left(x_{a},y_{a}\right)$. A larger $\vec{F_{a}}$ value indicates a higher current risk, and the direction of $\vec{F_{a}}$ is the same as that of $\vec{E_{a}}$. In summary, the driving risk field model is expressed as follows:


$$\begin{array}{c} \begin{array}{c} \vec{E}=\vec{E_{X\_a}}+\vec{E_{D\_a}}+\vec{E_{S\_a}}=\sum_{i=1}^{m}\vec{E_{X\_ia}}+\sum_{i=1}^{n}\vec{E_{D\_ia}}+\sum_{i=1}^{l}\vec{E_{S\_ia}}\\ \vec{F_{a}}=\vec{E}R_{a}M_{a}exp\left[-k_{\text{3}}v_{a}cos\left(\theta_{ab}\right)\right]\left(1+D_{ra}\right) \end{array}\} \end{array}$$ (7)

Where, $\vec{E}$ – resultant vector of the driving risk field intensity of vehicle a;

$\vec{E_{X\_a}}$、 $\vec{E_{X\_ia}}$ – resultant vector of behavioral field intensity of vehicle a at point $\left(x_{a},y_{a}\right)$;

$\vec{E_{D\_a}}$、 $\vec{E_{D\_ia}}$ – resultant vector of kinetic energy field intensity of vehicle a at point $\left(x_{a},y_{a}\right)$;

$\vec{E_{S\_a}}$、 $\vec{E_{S\_ia}}$ – resultant vector of potential energy field intensity of vehicle a at point $\left(x_{a},y_{a}\right)$.

The total field intensity vector of the driving risk field is the sum of the field intensity vectors of the behavioral field, kinetic energy field, and potential energy field. The magnitude of the driving risk field force acting on vehicle a can be calculated using the formula (2) in Formula (7).

## 4. Discussion and conclusion

### 4.1. Discussion

When vehicle a is located in the traffic flow, and other vehicles are numbered, the field intensity generated by other vehicles on vehicle a is as follows:


$$\begin{array}{c} \vec{E_{i\_a}}=\frac{k_{i}R_{a}M_{a}}{{\left|x_{i}-x_{a},y_{i}-y_{a}\right|}^{k_{\text{2}}}}\cdot \frac{\left(x_{i}-x_{a},y_{i}-y_{a}\right)}{\left|x_{i}-x_{a},y_{i}-y_{a}\right|}exp\left[k_{\text{3}}v_{a}cos\left(\theta_{ai}\right)\right]*\left(1+D_{ri}\right) \end{array}$$ (8)

Vehicle a is influenced by the comprehensive field intensity of the kinetic energy field, behavioral field, and potential energy field in the complex traffic flow environment. Where each vehicle-driver pair acts as an integrated risk source, emitting both kinetic and behavioral fields in the urban tunnel traffic environment, the driving risk field force acting on vehicle a is expressed as follows:


$$\begin{array}{c} \begin{array}{c} \vec{E_{i\_a}}=\frac{k_{i}R_{a}M_{a}}{{\left|x_{i}-x_{a},y_{i}-y_{a}\right|}^{k_{\text{2}}}}\cdot \frac{\left(x_{i}-x_{a},y_{i}-y_{a}\right)}{\left|x_{i}-x_{a},y_{i}-y_{a}\right|}exp\left[k_{\text{3}}v_{a}cos\left(\theta_{ai}\right)\right]*\left(1+D_{ri}\right)\\ i=1,2,3,\cdot \cdot \cdot ,n\\ E_{S\_cebi}=\frac{k_{\text{1}}R_{cb}M_{cb}}{r_{cb}^{k_{\text{2}}}}\\ E=E_{i\_a}+E_{S\_cebi}\\ F=EM_{a}R_{a}exp\left[-k_{\text{3}}v_{a}cos\left(\theta_{a}\right)\right]\left(1+D_{ra}\right) \end{array}\} \end{array}$$ (9)

Where, $E_{D\_ba}$ is the field intensity at vehicle a generated by the kinetic energy field of vehicle b;

$E_{X\_ba}$ is the field intensity at vehicle a generated by the behavioral field of the driver of vehicle b;

$E_{S\_cebi}$ is the potential energy field induced by the tunnel wall at the location of vehicle a;

$R_{a}$, $R_{b}$, $R_{cb}$ denote the road influence factors at vehicle a, vehicle b, and the tunnel wall, respectively;

$M_{a}$, $M_{b}$, $M_{cb}$ represent the equivalent masses of vehicle a, vehicle b, and the tunnel wall, respectively;

$D_{ra}$, $D_{rb}$ represent the risk factors associated with the driver of vehicle a and vehicle b, respectively;

F denotes the resultant force acting on vehicle a.

That is, the driving risk field force acting on vehicle a is the comprehensive vector superposition of the kinetic energy fields and behavioral fields generated by all surrounding traffic flow vehicles and their drivers, as well as the potential energy fields generated by factors such as tunnel sidewalls and road alignment.

Upon entering and traversing the tunnel, the vehicle’s path is divided into three distinct segments: the tunnel entrance transition zone, the interior section, and the tunnel exit transition zone. This study employs a six-lane urban underpass tunnel as the prototype for analysis. The spatial configuration is as follows: the segment from 0 m to 100 m represents the approach section preceding the tunnel entrance; from 100 m to 200 m corresponds to the entrance transition zone, where the longitudinal slope increases from 0% to 3%; the interior tunnel section spans from 200 m to 800 m, with the slope gradually decreasing from 3% to 0%; the exit transition zone extends from 800 m to 900 m, maintaining a constant 0% slope; and the section from 900 m to 1000 m constitutes the downstream segment after exiting the tunnel, as illustrated in Fig 7. For the coordinate system, the x-axis is defined along the centerline of the middle lane, representing the longitudinal direction of travel through the tunnel; the y-axis denotes the lateral displacement relative to the tunnel sidewall; and the z-axis quantifies the field strength of the potential energy field.

**Fig 7:**
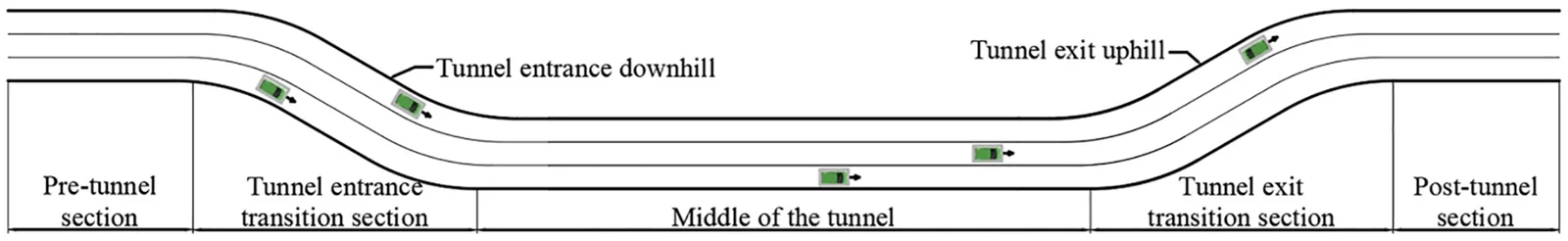
Urban Tunnel Diagram.

This study analyzes six application scenarios defined by three tunnel travel conditions (entrance transition, interior, exit transition) using the proposed risk field model:

(1) both vehicles in the same lane, entrance section (Fig 8a);
(2) vehicles in different lanes, entrance section (Fig 8b);
(3) both vehicles in the same lane, tunnel interior (Fig 9a);
(4) vehicles in different lanes, tunnel interior (Fig 9b);
(5) both vehicles in the same lane, exit section (Fig 10a);
(6) vehicles in different lanes, exit section (Fig 10b).

**Fig 8:**
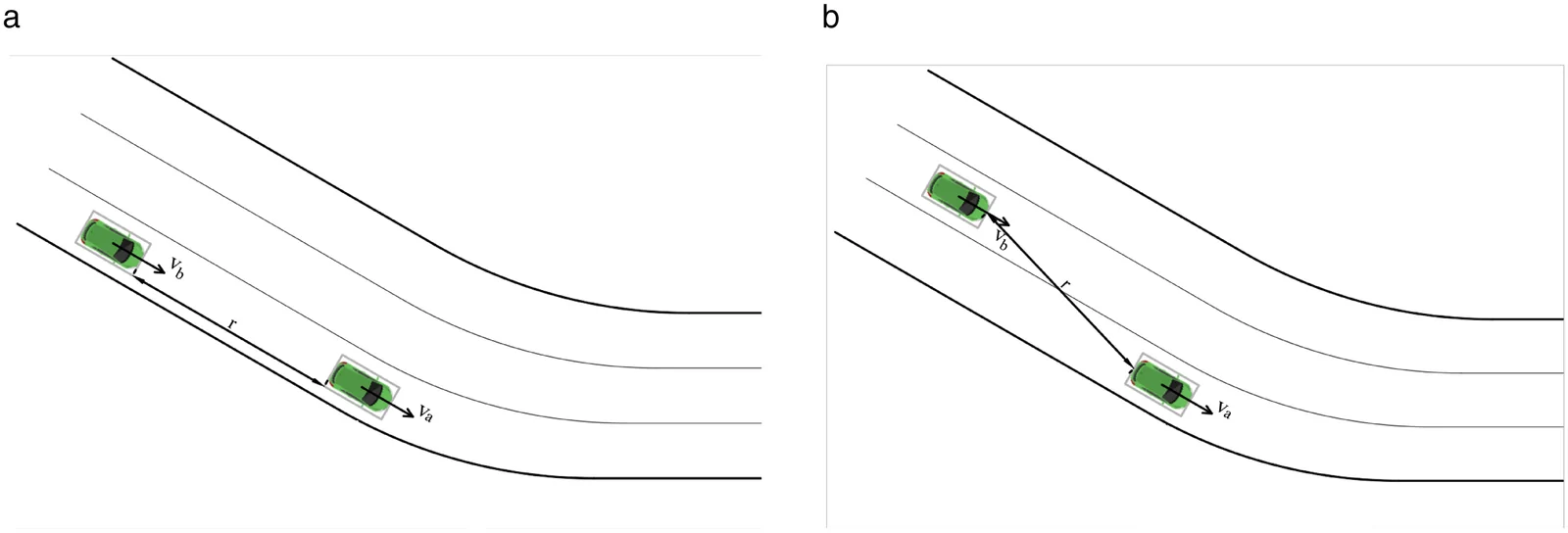
Urban tunnel entrance transition section scene. (a) Same lane. (b) Different lanes.

**Fig 9:**
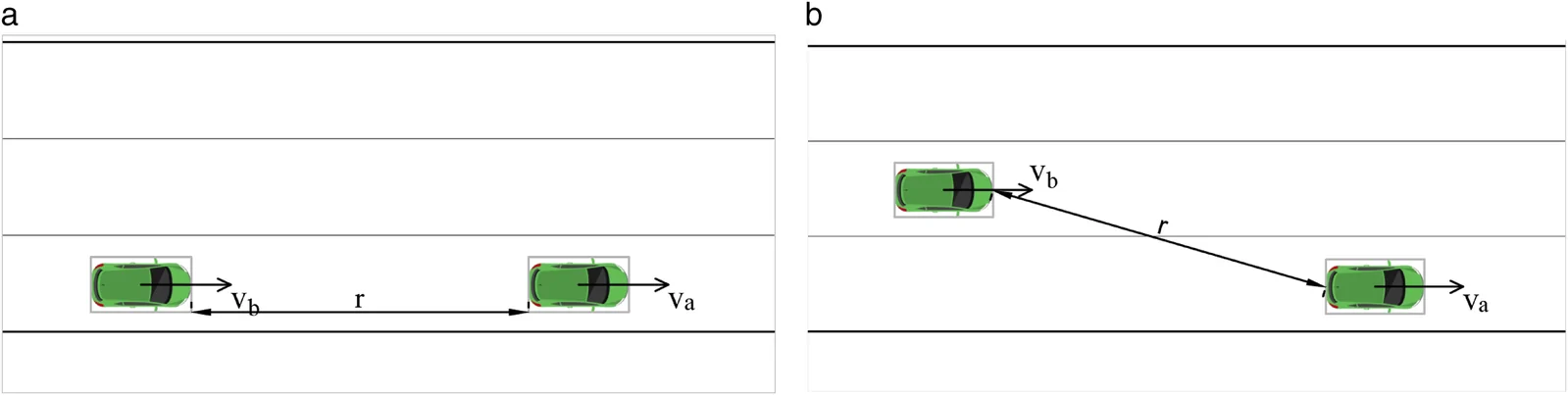
Interior scene of urban tunnel. (a) Same lane. (b) Different lanes.

**Fig 10:**
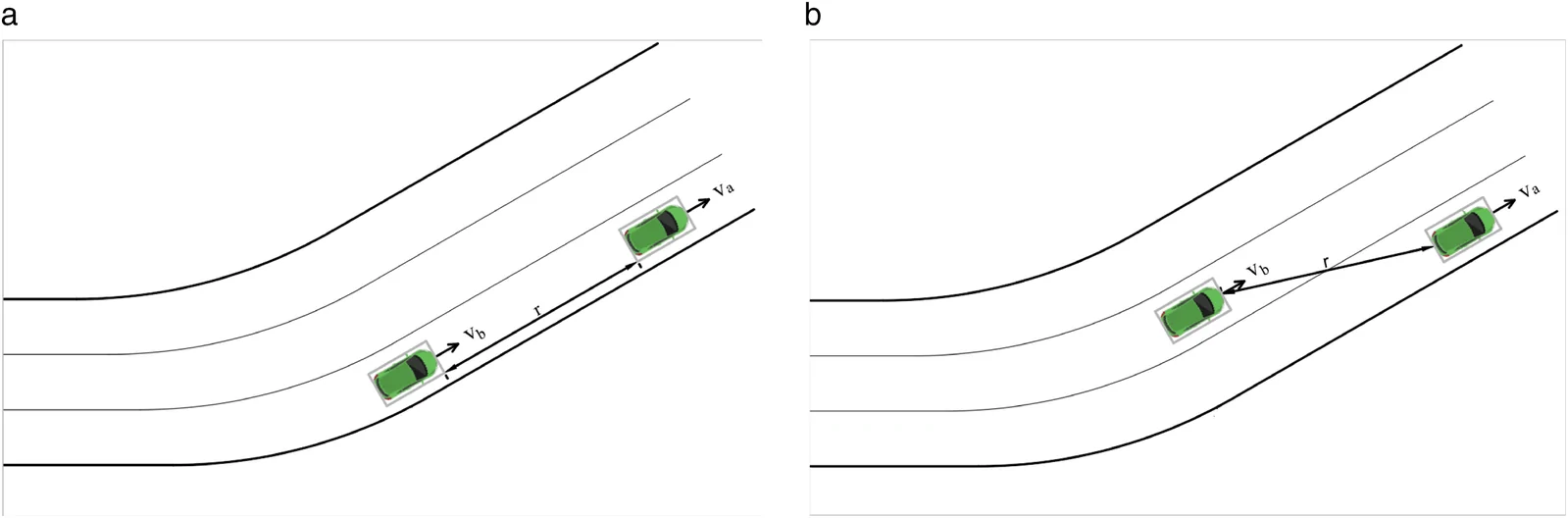
Urban tunnel exit transition section scene. (a) Same lane. (b) Different lanes.

Both vehicle a and vehicle b travel forward along the road centerline, with corresponding speeds $v_{a}$ and $v_{b}$, and the straight-line distance between the two vehicles is r. With both vehicles adhering to the central path, the model omits the potential energy contribution from the lane lines themselves. The driving risk of vehicle a is influenced by the tunnel sidewall, longitudinal slope gradient, and vehicle b. The driving risk generated by vehicle b is composed of two parts: the kinetic energy field formed by vehicle b itself and the behavioral field generated by the driver of vehicle b.

According to Formula (9), the driving risk field force acting on vehicle a in the three application scenarios is calculated. The model assumes that vehicle a travels at a constant speed of 60 km/h, i.e., $v_{a}=60km/h$. Set constant coefficients $k_{\text{1}}=0.001,k_{\text{2}}=1,k_{\text{3}}=0.05$, vehicle mass $M_{a}=2000kg,M_{b}=2000kg$, sidewall equivalent $M_{cb}=5000kg$. Due to behavioral adaptations to illumination shifts at the tunnel portal, driver reaction time is assigned as 2 s at the entrance and 1 s in the interior, respectively.

Fig 8 discusses the driving risk field force acting on vehicle a when both vehicles are located in the tunnel entrance transition section. Assume the tunnel entrance has a downhill slope of 3%. Vehicle a is located at the end point of the tunnel entrance, where the slope is 0%. At this time, the potential energy field at the location of vehicle a is a constant value. Vehicle b moves from the starting point position of the tunnel entrance. As the vehicle moves, the road slope value changes accordingly, i.e., the road influence factor changes with the spacing r between the two vehicles. Using the speed $v_{b}$ and the distance r as variables, the relationship between the risk field force acting on vehicle a and the speed $v_{b}$ of vehicle b and the distance r between them is calculated. Fig 11 shows that when vehicle b is closer to vehicle a, the force acting on vehicle a is greater; when vehicle b is traveling behind vehicle a, a higher speed of vehicle b results in a greater force on vehicle a; when vehicle b approaches vehicle a from the same lane, the force generated on vehicle a is significantly greater than when vehicle b approaches vehicle a from a different lane.

**Fig 11:**
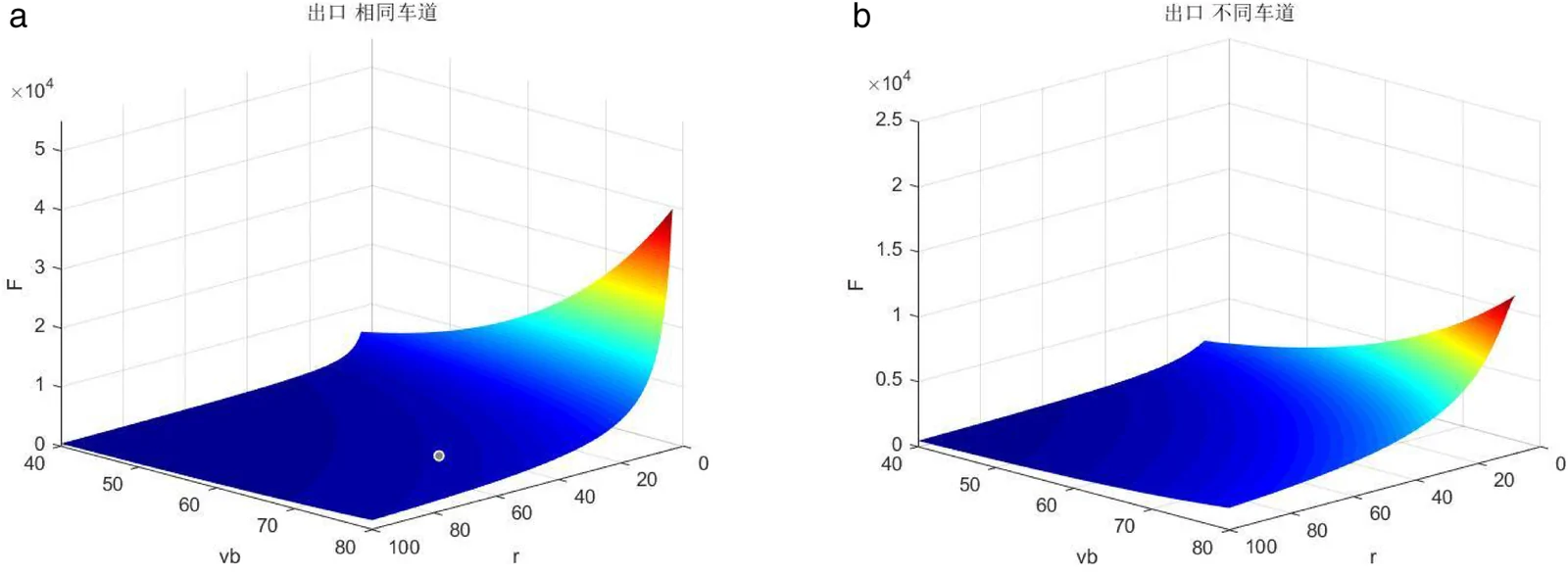
Driving risk field forces on vehicle a in tunnel entrance transition section. (a) Traveling in the same lane. (b) Traveling in different lanes.

Fig 9 discusses the magnitude of the driving risk field force acting on vehicle a when both vehicles are located inside the tunnel. When vehicles travel along the road centerline inside the tunnel, the road slope is 0, i.e., assume the road influence factor $R_{a}=1$, and the potential energy field intensity generated by the tunnel sidewall on the vehicle is a fixed value. The road slope remains 0 during vehicle b travel inside the tunnel. Using the travel speed $v_{b}$ of vehicle b and the distance r between the two vehicles as variables, the relationship between the risk field force acting on vehicle a and the speed $v_{b}$ of vehicle b and the distance r between them is calculated. Fig 12 shows that when the distance r between the two vehicles is smaller, the force acting on vehicle a is greater; when the travel speed of vehicle b is greater, the force on vehicle a is greater; when vehicle b and vehicle a travel in the same lane, the force generated on vehicle a is larger.

**Fig 12:**
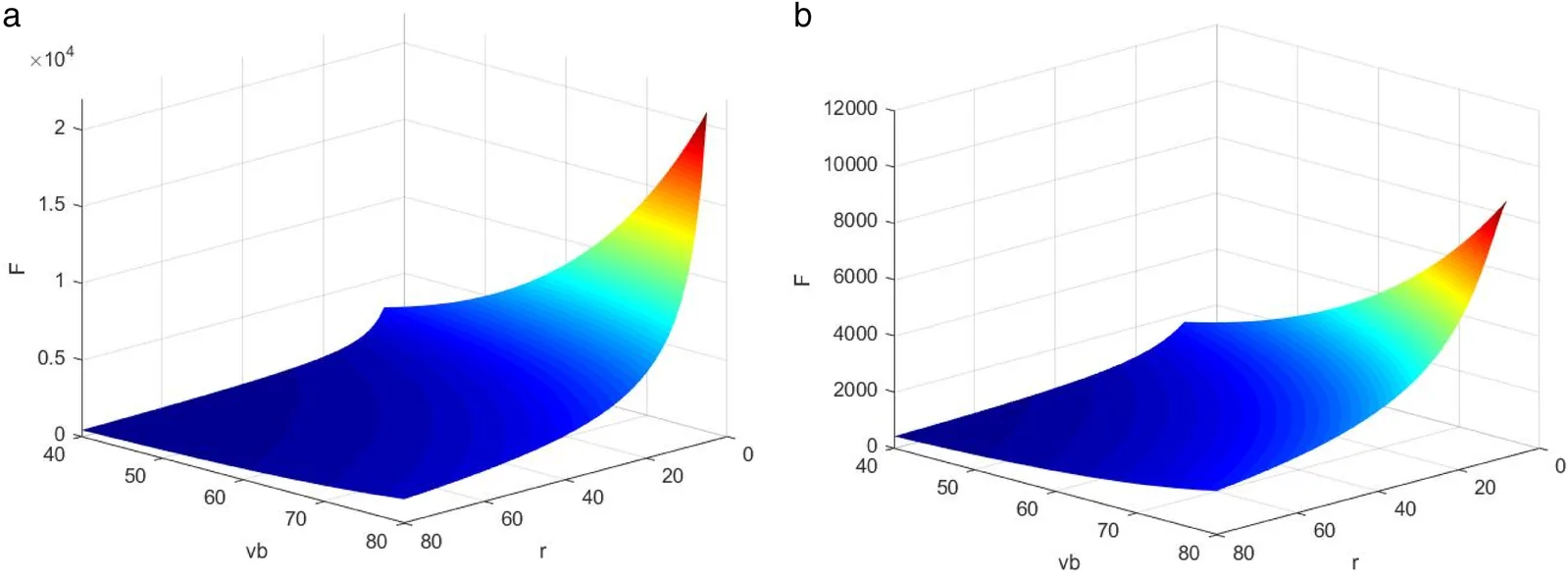
Driving risk field forces on vehicle a in tunnel. (a) Traveling in the same lane. (b) Traveling in different lanes.

Fig 10 discusses the magnitude of the driving risk field force acting on vehicle a when both vehicles are located in the tunnel exit transition section. Assume the tunnel exit has an uphill slope of 3%. Vehicle b moves from the starting point position of the tunnel exit. As the vehicle moves, the road slope value transitions gradually from 0 to 3%, i.e., the road influence factor changes with the spacing r between the two vehicles. Using the speed $v_{b}$ and the distance r as variables, the relationship between the risk field force acting on vehicle a and the speed $v_{b}$ of vehicle b and the distance r between them is calculated as shown in Fig 13. The same trend can be observed from the figure, i.e., the force acting on vehicle a is inversely proportional to the inter-vehicle spacing; the greater the speed of vehicle b, the greater the driving risk field force acting on vehicle a.

**Fig 13:**
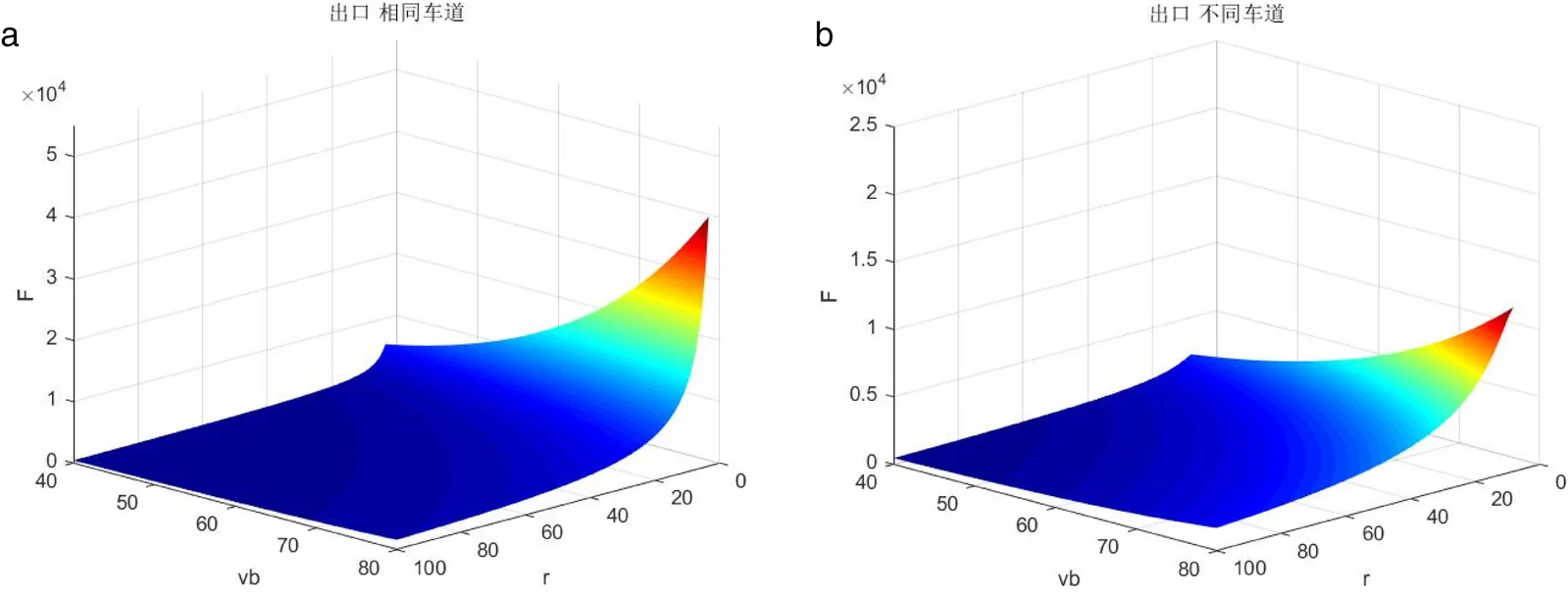
Driving risk field forces on vehicle a in tunnel exit transition section. (a) Traveling in the same lane. (b) Traveling in different lanes.

A comparative analysis of the tunnel zones (Figs 11–13) reveals a consistent hierarchy of risk field forces, independent of lane alignment: entrance transition > exit transition > interior. The maximum value of the risk field force experienced by vehicles at the entrance and exit sections of the tunnel is approximately 1.5 to 2.5 times that of the vehicles at the central section of the tunnel.

Further ranking by lane position and zone shows the force magnitude descends as follows: same-lane travel on the entrance downgrade, same-lane on the exit upgrade, same-lane in the interior, different-lanes on the entrance downgrade, different-lanes on the exit upgrade, and finally different-lanes in the interior. This hierarchy confirms that the rear-end collision risk inherent to same-lane travel substantially exceeds the lateral collision risk associated with different-lane scenarios.

### 4.2. Conclusion

This study substantiates the objective presence of high-risk driving zones within urban tunnels and elucidates the underlying mechanisms governing their formation. A driving risk field model for urban tunnels, grounded in multi-field theory, has been developed. The principal findings are summarized as follows:

(1) Under identical operational parameters, the kinetic energy field intensity within the tunnel entrance transition zone exceeds that observed in the interior section. Furthermore, the behavioral field intensity demonstrates a consistent trend across the three tunnel segments—entrance transition, interior, and exit transition. Under equivalent variable conditions, the behavioral field intensity follows a descending order: entrance transition segment > exit transition segment > tunnel interior.
(2) At the tunnel portal position, due to the superimposed influence of the longitudinal slope and tunnel sidewall, the corresponding potential energy field intensity is the maximum;
(3) Under the same conditions, the driving risk field force acting on the vehicle traveling in the same lane on the tunnel entrance downhill is the greatest, followed sequentially by the vehicle traveling in the same lane on the tunnel exit uphill, the vehicle traveling in the same lane inside the tunnel, the vehicle traveling in different lanes on the tunnel entrance downhill, the vehicle traveling in different lanes on the tunnel exit uphill, and the smallest is the vehicle traveling in different lanes inside the tunnel.

This paper develops a risk field model for urban tunnel traffic by drawing on multi-field theory. In the construction of the potential energy field component of the model, primary consideration is given to the tunnel’s longitudinal gradient and side walls. Moving forward, additional elements—including the central divider at tunnel entrances and exits, as well as tunnel auxiliary facilities—will be incorporated to further enhance the model’s precision. Furthermore, the application of the driving risk field model within the domains of autonomous driving and intelligent connectivity remains to be further explored.

## Supporting information

S1 File Data1. (TXT)

S2 File Data2. (TXT)

S3 File Data3. (TXT)

## References

[1] Lee J, Kirytopoulos K, Pervez A, Huang H. Understanding drivers’ awareness, habits and intentions inside road tunnels for effective safety policies. Accid Anal Prev. 2022;172:106690. doi: 10.1016/j.aap.2022.106690 35533421

[2] Jung S, Qin X. A data-driven approach to strengthening policies to prevent freeway tunnel strikes by motor vehicles. Accid Anal Prev. 2021;157:106171. doi: 10.1016/j.aap.2021.106171 33975092

[3] Wu R, Li L, Shi H, Rui Y, Ngoduy D, Ran B. Integrated driving risk surrogate model and car-following behavior for freeway risk assessment. Accid Anal Prev. 2024;201:107571. doi: 10.1016/j.aap.2024.107571 38608507

[4] Yan Y, Dai Y, Li X, Tang J, Guo Z. Driving risk assessment using driving behavior data under continuous tunnel environment. Traffic Inj Prev. 2019;20(8):807–12. doi: 10.1080/15389588.2019.1675154 31738591

[5] Tan H, Lu G, Liu M. Risk field model of driving and its application in modeling car-following behavior. IEEE Trans Intell Transport Syst. 2022;23(8):11605–20. doi: 10.1109/tits.2021.3105518

[6] Wan L, Yan Y, Zhang C, Liu C, Mao T, Wang W. Characteristics and identification of risky driving behaviors in expressway tunnels based on behavior spectrum. International Journal of Transportation Science and Technology. 2024;16:5–17. doi: 10.1016/j.ijtst.2023.10.006

[7] Chen S, Cheng K, Yang J, Zang X, Luo Q, Li J. Driving behavior risk measurement and cluster analysis driven by vehicle trajectory data. Appl Sci. 2023;13:5675.

[8] Qi W, Shen B, Wang L. Model of driver’s eye movement and ECG index under tunnel environment based on spatiotemporal data. John Wiley and Sons; 2020.

[9] Wang J, Huang H, Li Y, Zhou H, Liu J, Xu Q. Driving risk assessment based on naturalistic driving study and driver attitude questionnaire analysis. Accid Anal Prev. 2020;145:105680. doi: 10.1016/j.aap.2020.105680 32707185

[10] Xing Y, Lu J, Lu L, Jiang C, Cai X. Traffic safety evaluation model of road tunnels based on VISSIM. 2013. doi: 10.1061/9780784413159.157

[11] Ma Y, Lu L, Lu JJ. Safety evaluation model of urban cross-river tunnel based on driving simulation. Int J Inj Contr Saf Promot. 2017;24(3):293–302. doi: 10.1080/17457300.2016.1170044 27165860

[12] Cai X, Li Z, Qiao W, Cheng X, Peng B, Zhang D. Research on road traffic safety risk assessment based on the data of radar video integrated sensors. PROMTT. 2025;37(2):523–45. doi: 10.7307/ptt.v37i2.777

[13] Ma Y, Qi S, Fan L, Lu W, Chan C-Y, Zhang Y. Dynamic Bayesian network approach to evaluate vehicle driving risk based on on-road experiment driving data. IEEE Access. 2019;7:135050–62. doi: 10.1109/access.2019.2941959

[14] Xiong X, Chen L, Liang J. Vehicle driving risk prediction based on markov chain model. Discrete Dynamics in Nature and Society. 2018;2018:1–12. doi: 10.1155/2018/4954621

[15] Liu X, Lu J, Chen X, Fong YHC, Ma X, Zhang F. Attention based spatio-temporal graph convolutional network with focal loss for crash risk evaluation on urban road traffic network based on multi-source risks. Accid Anal Prev. 2023;192:107262. doi: 10.1016/j.aap.2023.107262 37598458

[16] Bao J, Liu P, Ukkusuri SV. A spatiotemporal deep learning approach for citywide short-term crash risk prediction with multi-source data. Accid Anal Prev. 2019;122:239–54. doi: 10.1016/j.aap.2018.10.015 30390519

[17] Mussah AR, Adu-Gyamfi Y. Machine learning framework for real-time assessment of traffic safety utilizing connected vehicle data. Sustainability. 2022;14(22):15348. doi: 10.3390/su142215348

[18] Zhu J, Ma Y, Lou Y. Multi-vehicle interaction safety of connected automated vehicles in merging area: A real-time risk assessment approach. Accid Anal Prev. 2022;166:106546. doi: 10.1016/j.aap.2021.106546 34965492

[19] Ding N, Jiao N, Zhu S, Liu B. Structural equations modeling of real-time crash risk variation in car-following incorporating visual perceptual, vehicular, and roadway factors. Accid Anal Prev. 2019;133:105298. doi: 10.1016/j.aap.2019.105298 31557617

[20] Li L, Gan J, Ji X, Qu X, Ran B. Dynamic driving risk potential field model under the connected and automated vehicles environment and its application in car-following modeling. IEEE Trans Intell Transport Syst. 2022;23(1):122–41. doi: 10.1109/tits.2020.3008284

[21] Yang Y, Huang H, Li G, Han B, Yuan Z, Ma H. A systematic review of resilience assessment and enhancement of urban integrated transportation networks. J Transport Geography. 2025;129:104420. doi: 10.1016/j.jtrangeo.2025.104420

[22] Yan X, Li G, Wu L, Cheng W, Zhao L, Min H. Modeling and analysis of mandatory merging decision behavior considering temporal and spatial risks. Transportation Research Record: Journal of the Transportation Research Board. 2025;2679(5):734–52. doi: 10.1177/03611981241308862

[23] Xie X, Li G, Wu L, et al. Investigation of freeway incident duration using classification and regression trees based on multisource data. Sensors. 2024; 24(22):7225. 10.3390/s24227225 PMC11598282 39599001

[24] Hao Y, Li G, Lu J, Cheng W, Yuan Q, Yao Z. Knowledge graph-based safety risk evaluation method for hazardous behaviors of road transport vehicles. Traffic Inj Prev. 2026;27(5):604–12. doi: 10.1080/15389588.2025.2540554 40833193

[25] Pan Y, Yang S, Wang C. Exploring the endogeneity between the autonomous vehicle takeover and crash severity: comparative analysis of structural equation modeling and generalized linear logit model. Traffic Injury Prevention. 2025: 1–8. 10.1080/15389588.2025.2492821 40815232

[26] Li G, Ji S, Cheng W, Wang W, Yan X, Yang Z, et al. A new framework for modelling traffic conflict interval time based on correlated random parameter duration model with heterogeneity in means and variances. Accid Anal Prev. 2026;224:108282. doi: 10.1016/j.aap.2025.108282 41166799

[27] Li Z, Xing G, Zhao X, Li H. Impact of the connected vehicle environment on tunnel entrance zone. Accid Anal Prev. 2021;157:106145. doi: 10.1016/j.aap.2021.106145 34020757

[28] Chang X, Li H, Rong J, Qin L, Zhao X. Spatiotemporal characteristics of vehicle trajectories in a connected vehicle environment—A case of an extra-long tunnel scenario. IEEE Systems Journal. 2021;15(2):2293–304. doi: 10.1109/jsyst.2020.2990650

